# Associations of triglyceride-glucose (TyG) index with chest pain incidence and mortality among the U.S. population

**DOI:** 10.1186/s12933-024-02209-y

**Published:** 2024-03-30

**Authors:** Yao Zhao, Yu Gu, Bili Zhang

**Affiliations:** 1 Department of Cardiovascular Surgery, Changhai Hospital, Naval Military Medical University, Shanghai, 200433 China; 2 Department of Neonatology, Women’s Hospital, Zhejiang University School of Medicine, Hangzhou, Zhejiang 310003 China; 3 Department of Cardiovasology, Changhai Hospital, Naval Military Medical University, 168 Changhai Road, Shanghai, 200433 China

**Keywords:** Triglyceride-glucose index, Insulin resistance, Chest pain, NHANES, Mortality

## Abstract

**Background:**

The triglyceride and glucose (TyG) index, a simple surrogate marker of insulin resistance, is related to cardiovascular disease. However, there is a lack of evidence for the relationship between the TyG index and chest pain. This study aimed to investigate the association of the TyG index with chest pain and to evaluate the relationship between the TyG index and all-cause mortality in participants with or without chest pain.

**Methods:**

The present study utilized data from the 2001–2012 National Health and Nutrition Examination Survey (NHANES), employing a combination of cross-sectional and cohort study designs. The association between the TyG index and chest pain was investigated using weighted logistic regression models. Weighted Cox proportional hazards models were used to estimate the hazard ratios (HRs) and 95% confidence intervals (95% CIs) for all-cause mortality. Restricted cubic spline analysis was used to explore linear or nonlinear relationships between the TyG index and chest pain or all-cause mortality.

**Results:**

The findings revealed a positive correlation between the TyG index and chest pain, even after adjusting for potential confounding factors (quartile 4 versus quartile 1, odds ratio [OR] 1.42, 95% confidence interval [CI] 1.14–1.77, *P* = 0.002). During a mean follow-up time of 139 months, a total of 2286 individuals (27.43%) experienced mortality. Weighted multivariate Cox regression models indicated that for each one-unit increase in the TyG index, the adjusted hazard ratio (HR) for mortality was 1.14 (95% CI = 0.94–1.37) for participants with chest pain and 1.25 (95% CI = 1.09–1.43) for those without chest pain. Furthermore, restricted cubic spline analysis revealed a linear relationship between the TyG index and chest pain (P for nonlinearity = 0.902), whereas a nonlinear relationship was shown between the TyG index and all-cause mortality among populations regardless of chest pain (all P for nonlinearity < 0.01).

**Conclusion:**

The TyG index was positively linked to a higher incidence of chest pain. Moreover, the TyG index was associated with all-cause mortality not only in participants with chest pain but also in those without chest pain.

**Supplementary Information:**

The online version contains supplementary material available at 10.1186/s12933-024-02209-y.

## Introduction

Chest pain is the second most common cause of emergency department visits in the United States after injury [1–3]. Moreover, chest pain also results in approximately 4 million outpatient visits annually [3]. Whether in the emergency room or on an outpatient basis, most patients with chest pain are hospitalized for further evaluation and treatment [4, 5]. In the United States, the lifetime incidence of chest pain is 20 to 40% [6]. There are a variety of causes of chest pain, ranging from benign to potentially life-threatening [5, 7–9]. Cardiovascular diseases (CVD), most of which can lead to chest pain, especially coronary artery disease, affect more than 18.2 million adults in the U.S. and are the leading cause of death for both men and women [10–12]. Hence, it is vital to identify the influencing factors associated with chest pain.

The triglyceride-glucose (TyG) index, a value calculated from fasting triglyceride and fasting blood glucose levels, has been recognized as a reliable, easily available, and less expensive biomarker of insulin resistance [13]. Prior studies have shown that a high TyG index is associated with a high risk of cardiovascular events such as hypertension, coronary artery stenosis, carotid atherosclerosis, arterial stiffness, stroke, coronary heart disease (CHD), and heart failure [14–20]. In addition, it has also been shown that the TyG index is associated with all-cause mortality in patients with a range of CVD [21–26]. However, chest pain, the most common concomitant symptom of CVD, has not yet been reported in any study.

Therefore, this study aimed to investigate the association of the TyG index with chest pain and to evaluate the relationship between the TyG index and all-cause mortality in participants with or without chest pain. The study population was drawn from the U.S. National Health and Nutrition Examination Survey (NHANES) for the period 2001–2012.

## Materials and methods

### Study design and participants

The NHANES program selects a representative sample of the U.S. population every two years using a sophisticated and complex method. Its main goal is to assess and evaluate the health and nutritional status of people in the U.S. To help ensure ethical standards, the survey was approved by the Institutional Review Board established by the National Center for Health Statistics Institutional Review Board. Furthermore, all those selected to participate willingly provided informed written consent prior to their inclusion in the study. The NHANES collects a wide range of information, including demographic information, response to questionnaires, medical examination data, and laboratory findings.

**Fig. 1:**
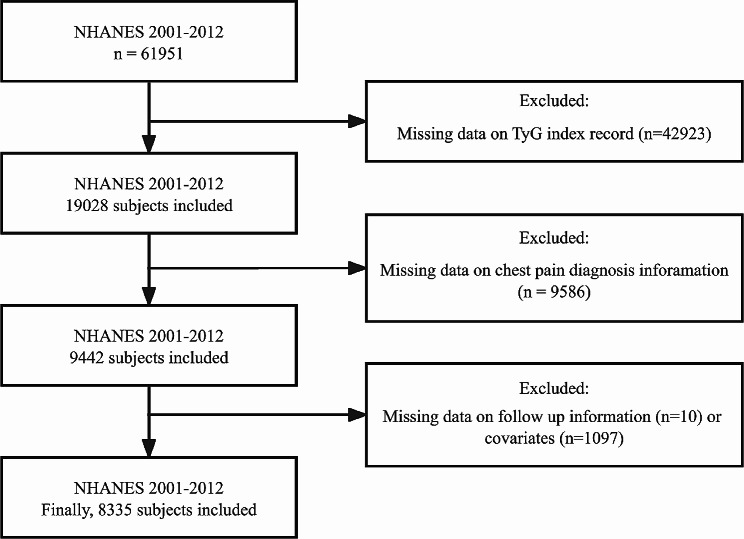
Flow chart of the study participants

In the NHANES 2001–2012 cycle, a total of 61,951 individuals participated in the study. After excluding subjects without information on the TyG index record, chest pain diagnosis data, covariates and follow-up data, the remaining sample was used for research analysis (Fig. 1).

### Exposure and outcome variables

In this study, the primary exposure variable was the TyG index. The TyG index was calculated according to the formula ln (fasting triglyceride mg/dL × fasting glucose mg/dL/2) [27]. The main outcomes were chest pain status and mortality. Chest pain was diagnosed if the participant answered “yes” to the inquiry “{Have you/Has SP} ever had any pain or discomfort in {your/her/his} chest?”. To investigate whether the TyG index is associated with the risk of all-cause mortality in patients with or without chest pain, we performed a survival analysis of the different groups. The participants’ endpoints and follow-up information were determined by linking their records to the National Death Index public access files through December 31, 2019 (https://www.cdc.gov/nchs/data-linkage/mortality.htm). We used the “MORTSTAT” variable as the status of death and the “PERMTH_EXM” variable as the follow-up time. The mean follow-up time was 143 ± 50 months for patients with a complaint of chest pain, while for participants without chest pain, it was 135 ± 49 months.

### Covariate

All the participants provided information on age, sex, race (classified as Mexican American, other Hispanic, non-Hispanic White, non-Hispanic Black, or other race), education level (classified as under high school, high school or equivalent, or above high school), family poverty-to-income ratio, and smoking status (classified as current smoker, former smoker or never smoker) through the survey questionnaires. Body mass index (BMI) was calculated using weight (kg)/height (m^2^) [28]. Laboratory results were obtained from serum specimens when the patients visited the mobile examination center, and the vials were stored under appropriate frozen conditions until they were shipped to the National Center for Environmental Health for testing. Congestive heart failure was diagnosed if the participant answered “yes” to the following question: “Has a doctor or other health professional ever told {you/SP} that {you/s/he} had congestive heart failure?”. CHD was diagnosed if the participant answered “yes” to the following question: “Has a doctor or other health professional ever told {you/SP} that {you/s/he} had CHD?”. Angina pectoris was diagnosed if the participant answered “yes” to the following question: “Has a doctor or other health professional ever told {you/SP} that {you/s/he} had angina, also called angina pectoris?”. Heart attack was diagnosed if the participant answered “yes” to the following question: “Has a doctor or other health professional ever told {you/SP} that {you/s/he} had a heart attack (also called myocardial infarction)?”. Stroke was diagnosed if the participant answered “yes” to the following question: “Has a doctor or other health professional ever told {you/SP} that {you/s/he} had a stroke?”. Hypertension was diagnosed if one of the following criteria was met: [1] the participant had a systolic blood pressure ≥ 140 mmHg or a diastolic blood pressure ≥ 90 mmHg; [2] the participant answered “yes” to the following question: “{Have you/Has SP} ever been told by a doctor or other health professional that {you/s/he} had hypertension, also called high blood pressure?”, or [3] the participant was currently using antihypertensive medication [29]. A diagnosis of CVD can be made if any of the above conditions are met. Participants were identified as having diabetes mellitus if they met any of the following criteria: had (a) a hemoglobin A1C concentration of 6.5%, (b) a fasting plasma glucose level of 126 mg/dL, (c) self-reported use of antidiabetic medications, or (d) a diagnosis of diabetes [30].

### Statistical analysis

In our study, “WTSAF2YR”, a sample weighting code for the fasting subsample from 2001 to 2012, was used as a weighted variable. Detailed information about the survey sample design and methods for calculating weights can be found in https://wwwn.cdc.gov/nchs/nhanes/analyticguidelines.aspx.

The baseline characteristics were divided into two groups according to the method used: one according to the presence or absence of chest pain and the other according to the quartile of the TyG index. Continuous variables are presented as the mean ± standard deviation (SD) and were compared by *t* test or Wilcoxon rank-sum test, as appropriate. Categorical variables are expressed as counts (weighted proportions) and were compared by the Pearson chi-square test.

The association between the TyG index and chest pain was estimated by weighted univariate and multivariate logistic regression analyses and presented as odds ratios (ORs) with 95% confidence intervals (CIs). The mortality rates among the different TyG index groups are shown as Kaplan–Meier curves and were compared by the log-rank test. The relationship between the TyG index and mortality in participants with or without chest pain was estimated by weighted univariate and multivariate Cox proportional hazards models in the form of hazard ratios (HRs) with 95% confidence intervals (CIs). The potential nonlinear relationships between the TyG index and chest pain and between the TyG index and all-cause mortality were further assessed using restricted cubic spline (RCS) curves based on multivariate logistic regression and Cox proportional hazards models. We used Akaike information criterion (AIC) to help determine the number of nodes in the RCS model. The lower the AIC index, the better the model. Therefore, we choose four nodes, which default to the 5%, 35%, 65% and 95% fixed percentiles of the TyG index distribution. To control for confounders, three weighted logistic regression and weighted Cox regression models were used: Model 1, unadjusted; Model 2, adjusted for age, sex and BMI; and Model 3, adjusted for age, sex, BMI, race, education levels, smoking status, total cholesterol, and hypertension, heart failure, coronary heart disease, angina, heart attack, stroke, antihyperlipidemic agents, and antidiabetic agents. Covariates in our models are variables that have been carefully selected based on their known associations with outcomes, as supported by existing scientific knowledge.

A two-sided *p* < 0.05 was considered to be statistically significant. All analyses were performed with R version 4.3.1 (R Foundation for Statistical Computing, Vienna, Austria).

## Results

### Baseline characteristics

A total of 8335 participants (4170 males and 4165 females) were enrolled in the present study. The baseline characteristics of the participants with and without chest pain are shown in Table 1. Approximately 28% (2344) of the participants in our study had chest pain. Specifically, a greater proportion of participants with chest pain had a lower educational attainment, a higher family poverty income ratio (PIR), a higher body mass index (BMI), and former and current smokers. In addition, participants with chest pain were more likely to have higher total cholesterol, triglyceride, hemoglobin type A1C (HbA1C), fasting glucose, and insulin levels, a higher TyG index, and a greater incidence of diabetes and CVD. The baseline characteristics of the total participants and participants with or without chest pain based on the TyG index are shown in Additional file 1: Tables S1, S2 and S3.

**Table 1:** Baseline characteristics of participants with or without chest pain

Characteristic	Overall (*n* = 8335)	No pain (*n* = 5991)	Pain (*n* = 2344)	*P*-value
Age, years	57 (12)	57 (12)	57 (12)	0.2
Sex				0.8
male	4,170 (48%)	3,001 (48%)	1,169 (48%)	
female	4,165 (52%)	2,990 (52%)	1,175 (52%)	
Race				0.14
Mexican American	1,325 (5.3%)	995 (5.6%)	330 (4.7%)	
Other Hispanic	553 (3.4%)	422 (3.3%)	131 (3.6%)	
Non-Hispanic White	4,431 (76%)	3,125 (76%)	1,306 (76%)	
Non-Hispanic Black	1,562 (9.8%)	1,102 (9.3%)	460 (11%)	
Other race	464 (5.4%)	347 (5.5%)	117 (5.1%)	
Education				< 0.001
Under high school	1,250 (7.4%)	876 (6.7%)	374 (9.2%)	
High school or equivalent	3,178 (35%)	2,264 (34%)	914 (38%)	
Above high school	3,907 (57%)	2,851 (59%)	1,056 (53%)	
Family PIR	3.22 (1.59)	3.30 (1.57)	3.00 (1.63)	< 0.001
BMI, kg/m^2^	29 (6)	29 (6)	30 (6)	< 0.001
Smoking status				< 0.001
Current smoker	1,595 (19%)	1,054 (18%)	541 (24%)	
Former smoker	2,722 (32%)	1,910 (31%)	812 (34%)	
Never smoker	4,018 (49%)	3,027 (51%)	991 (42%)	
Total cholesterol, mg/dL	204 (42)	205 (41)	201 (44)	< 0.001
Triglyceride, mg/dL	148 (136)	145 (141)	158 (124)	0.003
HbA1C, %	5.70 (0.92)	5.68 (0.87)	5.77 (1.05)	< 0.001
Fasting Glucose, mg/dL	108 (31)	107 (29)	110 (35)	0.001
Insulin, uU/mL	12 (12)	12 (10)	13 (15)	< 0.001
TyG index	8.78 (0.64)	8.75 (0.63)	8.85 (0.66)	< 0.001
TyG index group				< 0.001
Q1	2,084 (27%)	1,549 (28%)	535 (23%)	
Q2	2,084 (26%)	1,526 (26%)	558 (25%)	
Q3	2,083 (25%)	1,508 (25%)	575 (25%)	
Q4	2,084 (23%)	1,408 (21%)	676 (27%)	
CVD	1,339 (13%)	588 (7.4%)	751 (27%)	< 0.001
Heart failure	380 (3.5%)	143 (1.8%)	237 (8.1%)	< 0.001
CHD	529 (5.4%)	201 (2.7%)	328 (13%)	< 0.001
angina	363 (4.0%)	76 (1.1%)	287 (11%)	< 0.001
Heart attack	550 (5.4%)	173 (2.2%)	377 (14%)	< 0.001
Stroke	439 (4.0%)	242 (3.0%)	197 (6.5%)	< 0.001
Hypertension	1,669 (16%)	1,208 (16%)	461 (16%)	0.8
Diabetes mellitus	1,899 (17%)	1,289 (15%)	610 (19%)	< 0.001
Antihyperlipidemic agents	55 (0.5%)	32 (0.4%)	23 (0.7%)	0.2
Antidiabetic agents	193 (1.7%)	132 (1.7%)	61 (1.7%)	> 0.9

All values are presented as mean ± SD or as counts (weighted, proportion).

PIR: poverty income ratio; HbA1C: hemoglobin type A1C; CVD: cardiovascular disease; CHD: coronary heart disease

### Associations between the triglyceride-glucose index and chest pain

**Fig. 2:**
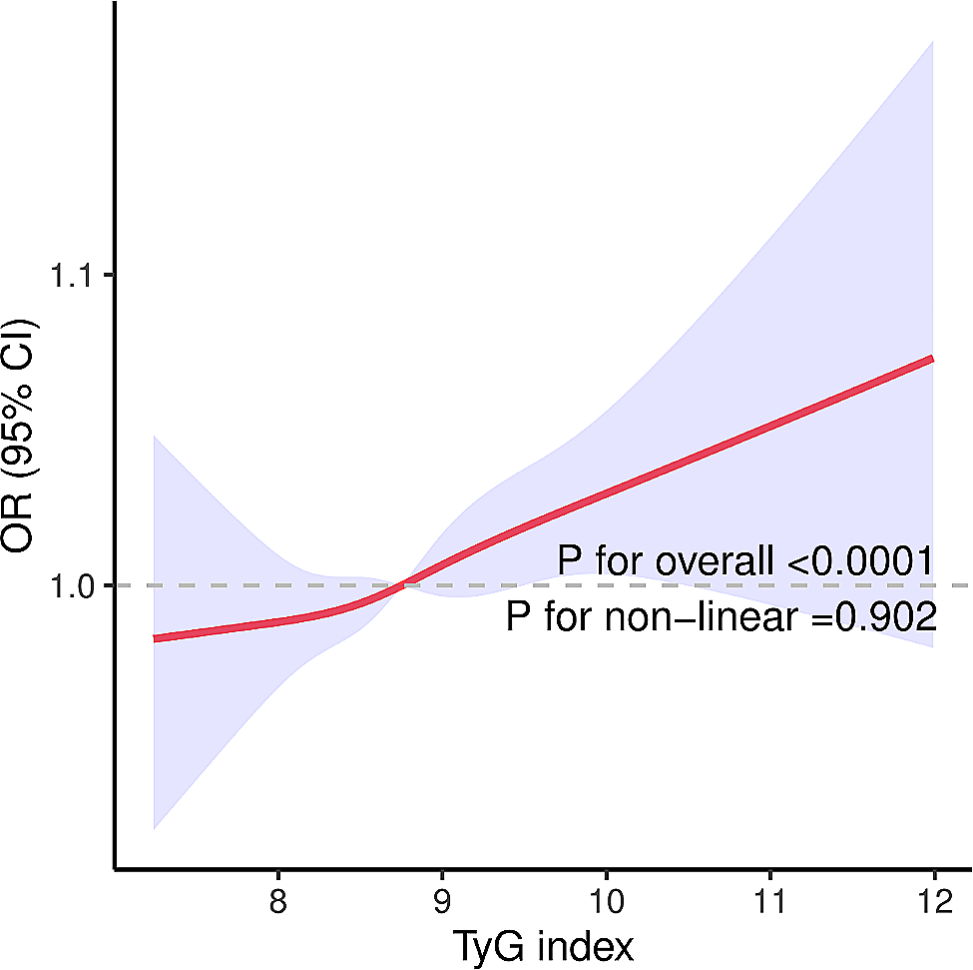
Restricted cubic spline curve for the association between the TyG index and the risk of chest pain. Red lines represent odds ratios, and blue areas represent 95% confidence intervals. The model was adjusted for age, sex, BMI, race, education, smoking status, total cholesterol, hypertension, heart failure, coronary heart disease, angina, heart attack, stroke, antihyperlipidemic agents, and antidiabetic agents

Among all enrolled participants, there was a significant positive association between the TyG index and chest pain. After adjusting for covariates, compared with that of the reference group (quartile 1), the risk of chest pain was increased by approximately 42% for participants in the fourth quartile (95% CI 1.14–1.77) of the TyG index (Table 2). When the TyG index was analyzed as a continuous predictor, it was also positively correlated with all-cause death; a 23% increase in the risk of mortality was associated with each one-unit increase in the TyG index (*P* < 0.001) (Table 2). Figure 2 shows restricted cubic splines indicating the dose‒response relationship between the TyG index and chest pain. A positive linear relationship was observed among all participants (P for nonlinearity = 0.902).

**Table 2:** ORs (95% CIs) for chest pain according to the TyG index

Characteristic	model 1			model 2			model 3		
	OR^1^	95% CI	*P*-value	OR^1^	95% CI	*P*-value	OR^1^	95% CI	*P*-value
Continuous	1.28	1.17, 1.40	< 0.001	1.23	1.12, 1.36	< 0.001	1.23	1.10, 1.39	< 0.001
TyG index									
Q1	—	—		—	—		—	—	
Q2	1.12	0.92, 1.35	0.3	1.08	0.89, 1.31	0.4	1.10	0.91, 1.35	0.3
Q3	1.18	0.98, 1.43	0.086	1.12	0.92, 1.36	0.3	1.13	0.91, 1.39	0.3
Q4	1.52	1.26, 1.84	< 0.001	1.42	1.16, 1.74	0.001	1.42	1.14, 1.77	0.002
P for trend			< 0.001			< 0.001			< 0.001

OR: odds ratio, CI: confidence interval

Model 1: Unadjusted

Model 2: Adjusted for age, sex, and BMI

Model 3: Adjusted for age, sex, BMI, race, education, smoking status, total cholesterol, hypertension, heart failure, coronary heart disease, angina, heart attack, stroke, antihyperlipidemic agents, and antidiabetic agents

**Fig. 3:**
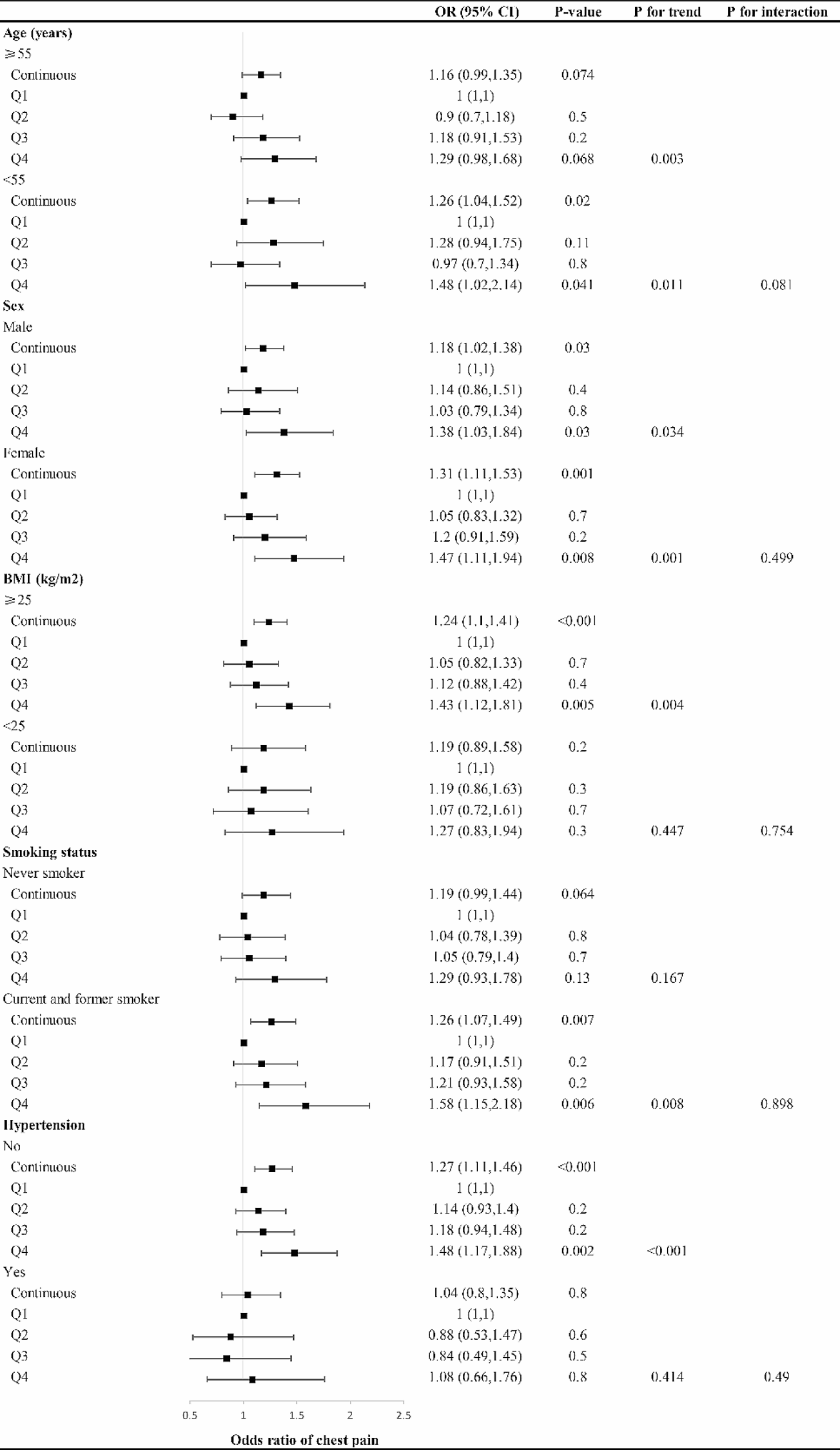
Subgroup analysis of the association between the TyG index and chest pain incidence. Adjusted for age, sex, BMI, race, education, smoking status, total cholesterol, hypertension, heart failure, coronary heart disease, angina, heart attack, stroke, antihyperlipidemic agents, and antidiabetic agents. OR: odds ratio, CI: confidence interval

Furthermore, the results of subgroup analyses between the TyG index and chest pain stratified by age, sex, BMI, smoking status, and hypertension incidence are presented in Fig. 3. It is clear that participants in the highest quartile of the TyG index in each subgroup had a greater risk of chest pain. The association persists when the TyG index is in continuous form.

### Correlation between the triglyceride-glucose index and all-cause mortality

**Fig. 4:**
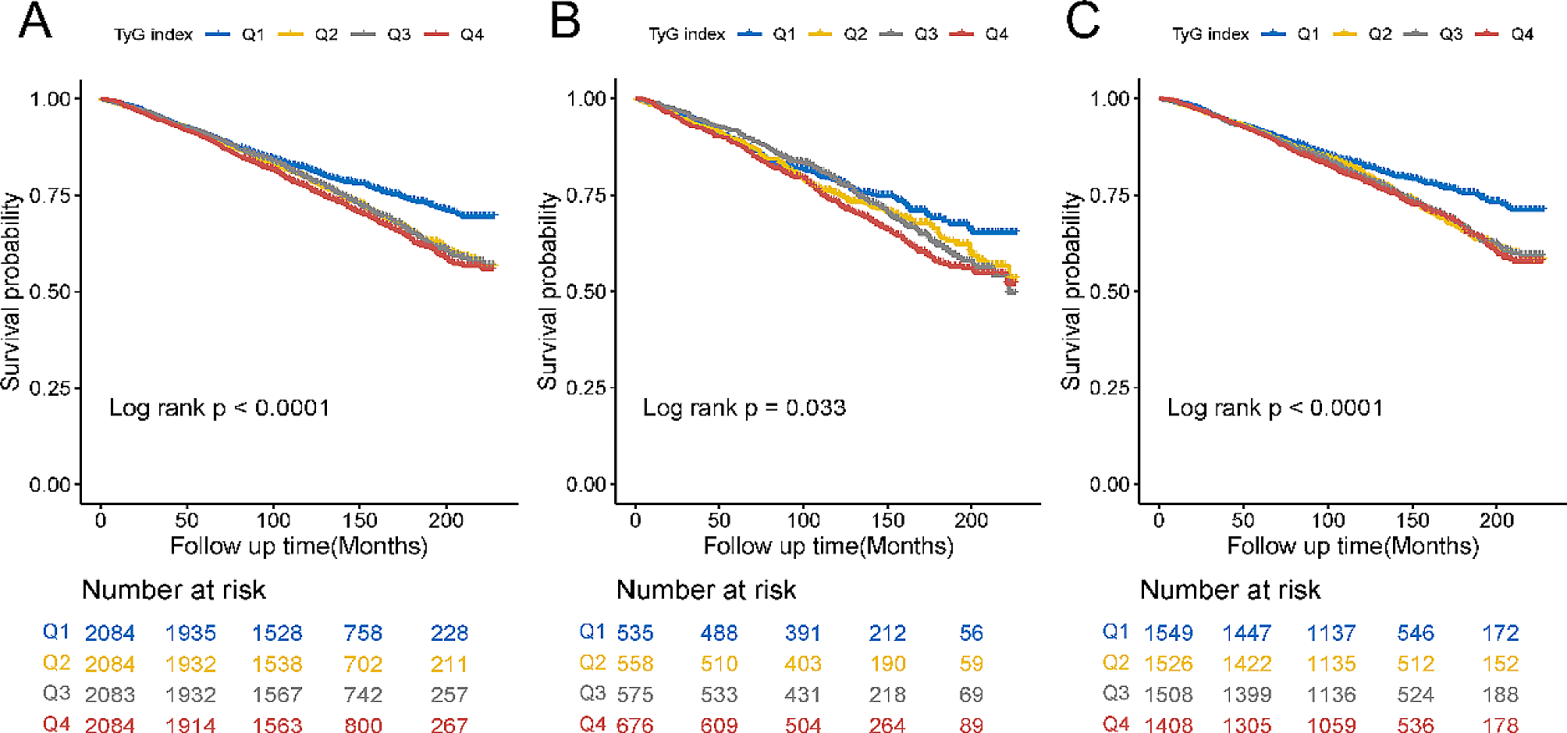
Kaplan–Meier analysis of all-cause mortality in (**A**) total participants, (**B**) participants with chest pain, and (**C**) participants without chest pain

**Fig. 5:**
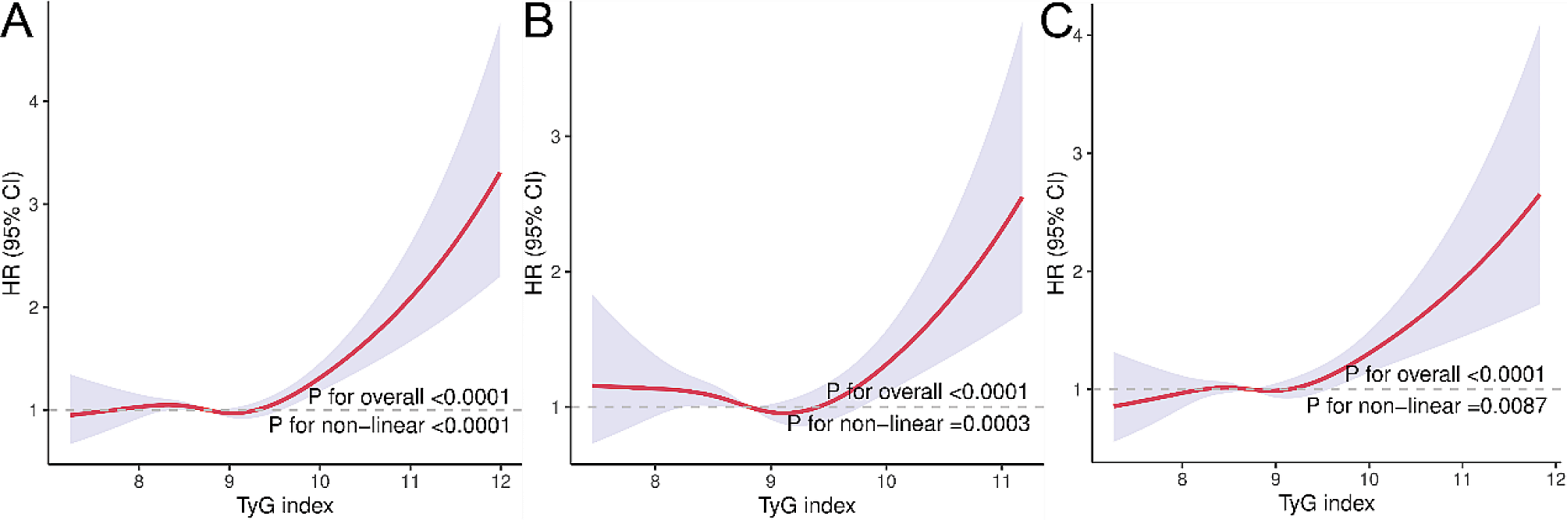
Restricted cubic spline curves for the association between the TyG index and all-cause mortality in (**A**) total participants, (**B**) participants with chest pain, and (**C**) participants without chest pain. Red lines represent references for hazard ratios, and blue areas represent 95% confidence intervals. The model was adjusted for age, sex, BMI, race, education, smoking status, total cholesterol, hypertension, heart failure, coronary heart disease, angina, heart attack, stroke, antihyperlipidemic agents, and antidiabetic agents

Among the total participants, 2286 individuals (27.43%) experienced mortality. Kaplan-Meier survival curves demonstrated that a higher TyG index was associated with greater all-cause mortality in all participants, regardless of whether they had chest pain (All log rank *p* value < 0.05, as depicted in Fig. 4A, B, C). Multivariate Cox regression models indicated that for each one-unit increase in the TyG index, the adjusted hazard ratios (HRs) for mortality were 1.21 (95% CI: 1.09–1.33) for the total participants, 1.14 (95% CI: 0.94–1.37) for the chest pain population and 1.25 (95% CI: 1.09–1.43) for the non-chest pain population (Table 3). The relationship between the TyG index and all-cause mortality was further assessed by RCS curves (Fig. 5). The RCS analysis revealed that the TyG index, as a continuous variable, was positively associated with an increased adjusted risk of all-cause mortality in patients with (p for nonlinear = 0.0003) chest pain and in the total enrolled patients (p for nonlinear < 0.0001). The analysis also revealed that high TyG index values were associated with an increased risk of death in the non-chest pain population (p for nonlinearity = 0.0087). Subgroup analysis was also in accordance with these findings (Fig. S1, S2, S3).

**Table 3:** HRs (95% CIs) for all-cause mortality according to the TyG index

Characteristic	model 1			model 2			model 3		
	HR^1^	95% CI^1^	*P*-value	HR^1^	95% CI^1^	*P*-value	HR^1^	95% CI^1^	*P*-value
**All participants**									
Continous	1.29	1.21, 1.37	< 0.001	1.19	1.08, 1.30	< 0.001	1.21	1.09, 1.33	< 0.001
TyG index									
Q1	—	—		—	—		—	—	
Q2	1.35	1.14, 1.60	< 0.001	0.98	0.84, 1.14	0.8	1.01	0.86, 1.18	> 0.9
Q3	1.48	1.26, 1.73	< 0.001	1.03	0.90, 1.18	0.7	1.05	0.91, 1.21	0.5
Q4	1.64	1.42, 1.89	< 0.001	1.18	1.02, 1.36	0.021	1.19	1.03, 1.37	0.021
P for trend			< 0.001			< 0.001			< 0.001
**Pain**									
Continous	1.18	1.02, 1.36	0.029	1.13	0.96, 1.34	0.13	1.14	0.94, 1.37	0.2
TyG index									
Q1	—	—		—	—		—	—	
Q2	1.14	0.82, 1.58	0.4	0.90	0.64, 1.26	0.5	0.90	0.65, 1.27	0.6
Q3	1.22	0.92, 1.61	0.2	0.88	0.67, 1.16	0.4	0.88	0.67, 1.15	0.3
Q4	1.45	1.09, 1.94	0.010	1.12	0.85, 1.48	0.4	1.13	0.84, 1.50	0.4
P for trend			0.065			0.065			0.065
**No pain**									
Continous	1.33	1.21, 1.46	< 0.001	1.20	1.05, 1.37	0.007	1.25	1.09, 1.43	0.002
TyG index									
Q1	—	—		—	—		—	—	
Q2	1.45	1.14, 1.83	0.002	1.02	0.83, 1.25	0.9	1.06	0.86, 1.32	0.6
Q3	1.60	1.27, 2.01	< 0.001	1.10	0.90, 1.35	0.4	1.15	0.93, 1.42	0.2
Q4	1.68	1.33, 2.13	< 0.001	1.18	0.94, 1.49	0.2	1.22	0.96, 1.54	0.10
P for trend			< 0.001			< 0.001			< 0.001

HR = Hazard Ratio, CI = Confidence Interval

Model 1: Unadjusted

Model 2: Adjusted for age, sex, and BMI

Model 3: Adjusted for age, sex, BMI, race, education, smoking status, total cholesterol, hypertension, heart failure, coronary heart disease, angina, heart attack, stroke, antihyperlipidemic agents, and antidiabetic agents

## Discussion

This study revealed the relationships between the TyG index and symptoms of chest pain and between the TyG index and all-cause mortality in subjects with or without chest pain utilizing data from NHANES 2001–2012. We found that the TyG index was linearly associated with chest pain and that patients with an elevated TyG index were at greater risk of chest pain. Furthermore, it is noteworthy that the effect of the TyG index, a simple surrogate marker of insulin resistance, on mortality was observed not only in patients with chest pain but also in those without.

Existing evidence suggests that metabolic factors play a crucial role in the development of various CVDs [31–33]. In particular, the TyG index, an indicator related to metabolism, has recently been proven to be associated with the risk of different types of CVD. Laura et al. first suggested that the TyG index is an independent risk factor for CVD events, including CHD, cerebrovascular disease and peripheral arterial disease, using a large population sample from the Vascular Metabolic CUN cohort (VMCUN cohort) with a median follow-up of 10 years [17]. Zheng et al. showed that a higher TyG index was associated with an increased risk of incident hypertension after a follow-up period of 9 years. According to the Hanzhong Adolescent Hypertension Cohort study, the highest TyG index trajectory was the most likely to lead to increased arterial stiffness after full adjustment [34]. Huang et al. conducted a prospective cohort study that revealed that a chronically elevated TyG index in hypertensive patients is linked to an increased risk of stroke, particularly ischemic stroke [35]. Xu et al. reported that the TyG index was positively correlated with the likelihood of heart failure [36]. Mendelian randomization analysis by Li et al. revealed that a higher TyG index is a direct cause of incident heart failure in the general population [37]. Wang et al. demonstrated that the TyG index is a reliable marker for predicting the extent and prognosis of multivessel coronary disease in patients with acute coronary syndrome [38]. Zhao and colleagues observed a positive correlation between the TyG index and the risk of CHD and the severity of coronary atherosclerosis among patients with non-alcoholic fatty liver disease [39].

Chest pain is the most common symptom of the cardiovascular system, but few studies have investigated the link between the TyG index and chest pain. In Brazil, a study showed that the TyG index was positively linked to an increased incidence of symptomatic coronary artery disease and could be utilized as an indicator of atherosclerosis. Furthermore, our study demonstrated that the TyG index was positively correlated with a higher incidence of chest pain. Compared to patients with the lowest TyG index, those with the highest TyG index had a greater incidence of chest pain. After fully adjusting, the relationship still remains and has become even more pronounced. Subgroup analysis was also in line with these findings.

The TyG index has been linked to mortality in various populations, as evidenced by previous studies. According to Sun et al., among middle-aged and elderly U.S. population, the TyG index had a U-shaped relationship with all-cause mortality, and the TyG index associated with the lowest risk of all-cause mortality was 9.18 [40]. Du et al. reported that the TyG index is connected to the risk of all-cause mortality in individuals who are obese [41]. Apart from the populations mentioned above, the TyG index has been studied more often in relation to mortality from CVDs. As reported by Pang and colleagues, the TyG index is a marker of mortality during the long-term follow-up of middle-aged and elderly hypertension patients [42]. A study from the Medical Information Mart for Intensive Care III (MIMIC-III) database demonstrated that the TyG index is a powerful and reliable indicator of higher mortality in critically ill patients with CHD [43]. Another study from the MIMIC-IV database has proven that the association between the TyG index and all-cause death is maintained in critically ill patients with ischemic stroke [44]. In addition, a study in China demonstrated that the TyG index was a significant predictor of mortality in patients with chronic heart failure [45]. Furthermore, two studies have demonstrated that the TyG index impacts clinical outcomes in patients with acute myocardial infarction, regardless of diabetes status [46, 47].

Our study revealed that the TyG index was positively associated with chest pain and was correlated with all-cause mortality in the chest pain group. Although the TyG index was linearly linked to the incidence of chest pain, it has a nonlinear relationship with all-cause mortality caused by chest pain. In addition to the chest pain population, the TyG index predicts all-cause mortality in individuals with non-chest pain. Furthermore, the relationship held even after all the adjustments were made. Subgroup analysis was also in accordance with these findings. Most intriguingly, our study provided evidence that even in the absence of chest pain, it is important to take this seriously. In the future, we can investigate the predictive role of the TyG index in predicting the prognosis of other non-CVD populations. Therefore, the scope of application of TyG as an emerging marker for CVD should be expanded.

The TyG index, a simple surrogate marker of insulin resistance, may be associated with chest pain and mortality through the following mechanisms. First, insulin resistance can result in either impaired vasodilation or increased vasodilation, leading to peripheral endothelial dysfunction [48–50]. Blood insulin levels in the normal range stimulate vasodilation at the arterial, venous and microcirculatory levels by increasing endothelial nitric oxide production [48, 51, 52]. Additionally, insulin resistance has been demonstrated to diminish the expression and function of the endothelial nitric oxide-synthase gene in both endothelial cells and microvessels within insulin resistant rats [53]. Second, insulin resistant may also exert significant influences on structural vascular adaptations, encompassing vascular remodeling, rarefaction, and collateralization [49]. Previous research has demonstrated a negative correlation between insulin resistance and vascular endothelial growth factor levels [54]. In addition, Bonner et al. observed that the loss of vascular endothelial growth factor in murine cardiac muscle leads to capillary rarefaction and contributes to insulin resistance [55]. Third, insulin resistance is correlated with heightened systemic inflammation [56]. IL-6 has been demonstrated to be linked with coronary microvascular dysfunction [57]. Additionally, IL-6 has been shown to impede the relaxation mediated by endothelium-dependent nitric oxide and augment contraction in an experimental model, indicating a direct involvement of IL-6 in microvascular dysfunction in the setting of insulin resistance [58]. The TyG index holds clinical significance as a representation of insulin resistance. To effectively manage the TyG index, our potential intervention measures involve controlling blood sugar levels, while preventive measures include improving diet and increasing physical activity. Moving forward, we aim to delve deeper into the molecular mechanisms underlying the impact of the TyG index on chest pain and mortality.

The advantage of our study is that the findings come from a large and representative sample of U.S. individuals, which has positive influences on the research. Nevertheless, our study also has certain limitations. First, despite adjusting for numerous confounding factors, the observed association between TyG index and chest pain may still be susceptible to unmeasured or residual confounders. Second, a causal relationship between the TyG index and chest pain could not be determined. In the future, prospective randomized controlled trials or Mendelian randomized studies will be imperative to further elucidate the causal relationship between TyG and the incidence and mortality of chest pain. Third, due to the inherent characteristics of the NHANES database questionnaire, inferring the etiology (episodic or persistent) and nature (cardiogenic or non-cardiogenic) of chest pain becomes challenging. In future studies, a more comprehensive investigation into the influence of the TyG index on different subtypes of chest pain can be conducted. Fourth, the NHANES database’s inherent limitations made it arduous for us to study the dynamic nature of associations between the TyG index and health outcomes. To fully interpret the dynamic nature of these associations, we intend to conduct prospective cohort studies in the future. Fifth, the limitation of our study to the U.S. population was due to the fact that we relied on data from the NHANES database, which exclusively collects information on the U.S. population. In order to further explain external validity and potential variations in different populations, we have planned a subsequent multi-center study. Sixth, even though the NHANES questionnaire adheres to strict quality control, the validity of the study may still be impacted by the limitations of self-reported measurement and potential misclassification of results. Finally, the study did not discuss the influence of the TyG index on cause-specific mortality in participants with or without chest pain.

## Conclusion

The TyG index was positively linked to a higher incidence of chest pain. However, the TyG index was associated with all-cause mortality not only in participants with chest pain but also in those without chest pain.

### Electronic supplementary material

Below is the link to the electronic supplementary material.

Supplementary Material 1

## Data Availability

The data used in this study are openly available from the Centers for Disease Control and Prevention at https://www.cdc.gov/nchs/nhanes/index.htm.

## Abbreviations

NHANES: National Health and Nutrition Examination Survey
MIMIC: Medical Information Mart for Intensive Care
SD: Standard deviation
PIR: Poverty income ratio
CVD: Cardiovascular disease
CHD: Coronary heart disease
BMI: Body mass index
HbA1c: Hemoglobin type A1C
RCS: Restricted cubic spline
Q: Quartiles
OR: Odds ratio
HR: Hazard ratio
CI: Confidence interval
AIC: Akaike information criterion
