# Supplementary material for: Associations of triglyceride-glucose (TyG) index with chest pain incidence and mortality among the U.S. population
Source: Cardiovasc Diabetol. 2024 Mar 30;23:111. doi: 10.1186/s12933-024-02209-y (PMC10981836; doi:10.1186/s12933-024-02209-y)
Supplement: Supplementary file 1 — Supplementary Material 1 [file 12933_2024_2209_MOESM1_ESM.docx]

**Additional files**

**Table S1** Baseline characteristics of all participants according to the TyG index

| **Characteristic** | **Overall** | **Q1 (n = 2084)** | **Q2 (n = 2084)** | **Q3 (n = 2083)** | **Q4(n = 2084)** | **P-value** |
| --- | --- | --- | --- | --- | --- | --- |
|  | **(n = 8335)** | **(6.79-8.36)** | **(8.37-8.75)** | **(8.76-9.18)** | **(9.19-13.40)** |  |
| Age, years | 57 (12) | 54 (12) | 57 (12) | 58 (12) | 58 (12) | <0.001 |
| Sex |  |  |  |  |  | <0.001 |
| male | 4,170 (48%) | 892 (38%) | 1,046 (49%) | 1,057 (49%) | 1,175 (57%) |  |
| female | 4,165 (52%) | 1,192 (62%) | 1,038 (51%) | 1,026 (51%) | 909 (43%) |  |
| Race |  |  |  |  |  | <0.001 |
| Mexican American | 1,325 (5.3%) | 200 (3.4%) | 293 (4.9%) | 349 (5.7%) | 483 (7.8%) |  |
| Other Hispanic | 553 (3.4%) | 105 (2.5%) | 127 (2.8%) | 176 (4.9%) | 145 (3.5%) |  |
| Non-Hispanic White | 4,431 (76%) | 1,064 (75%) | 1,120 (76%) | 1,143 (76%) | 1,104 (77%) |  |
| Non-Hispanic Black | 1,562 (9.8%) | 602 (15%) | 421 (10%) | 297 (7.3%) | 242 (5.5%) |  |
| Other race | 464 (5.4%) | 113 (4.1%) | 123 (5.8%) | 118 (5.8%) | 110 (6.1%) |  |
| Education |  |  |  |  |  | <0.001 |
| Under high school | 1,250 (7.4%) | 213 (5.1%) | 290 (6.9%) | 315 (8.1%) | 432 (10%) |  |
| High school or equivalent | 3,178 (35%) | 708 (29%) | 782 (35%) | 818 (37%) | 870 (42%) |  |
| Above high school | 3,907 (57%) | 1,163 (66%) | 1,012 (58%) | 950 (55%) | 782 (48%) |  |
| Family PIR | 3.22 (1.59) | 3.40 (1.58) | 3.27 (1.59) | 3.11 (1.58) | 3.05 (1.60) | <0.001 |
| BMI, kg/m^2^ | 29 (6) | 27 (6) | 29 (7) | 30 (6) | 31 (6) | <0.001 |
| Smoking status |  |  |  |  |  | <0.001 |
| Current smoker | 1,595 (19%) | 373 (17%) | 390 (18%) | 390 (21%) | 442 (22%) |  |
| Former smoker | 2,722 (32%) | 607 (29%) | 644 (30%) | 711 (33%) | 760 (36%) |  |
| Never smoker | 4,018 (49%) | 1,104 (54%) | 1,050 (51%) | 982 (47%) | 882 (42%) |  |
| Total cholesterol, mg/dL | 204 (42) | 193 (36) | 200 (36) | 207 (41) | 219 (50) | <0.001 |
| Triglyceride, mg/dL | 148 (136) | 68 (15) | 105 (16) | 150 (27) | 288 (226) | <0.001 |
| HbA1C, % | 5.70 (0.92) | 5.41 (0.47) | 5.54 (0.53) | 5.68 (0.67) | 6.25 (1.49) | <0.001 |
| Fasting Glucose, mg/dL | 108 (31) | 95 (11) | 102 (14) | 107 (19) | 130 (52) | <0.001 |
| Insulin, uU/mL | 12 (12) | 8 (7) | 11 (7) | 13 (14) | 18 (15) | <0.001 |
| TyG index | 8.78 (0.64) | 8.06 (0.24) | 8.56 (0.11) | 8.96 (0.12) | 9.66 (0.49) | <0.001 |
| CVD | 1,339 (13%) | 254 (9.0%) | 310 (11%) | 351 (14%) | 424 (17%) | <0.001 |
| Heart failure | 380 (3.5%) | 63 (2.3%) | 83 (2.6%) | 92 (3.7%) | 142 (5.6%) | <0.001 |
| CHD | 529 (5.4%) | 93 (3.8%) | 119 (4.7%) | 145 (6.3%) | 172 (7.2%) | <0.001 |
| angina | 363 (4.0%) | 52 (2.3%) | 89 (3.7%) | 102 (4.4%) | 120 (5.6%) | <0.001 |
| Heart attack | 550 (5.4%) | 100 (3.5%) | 131 (5.3%) | 135 (5.4%) | 184 (7.5%) | <0.001 |
| Stroke | 439 (4.0%) | 98 (3.3%) | 102 (3.6%) | 114 (4.4%) | 125 (4.8%) | 0.12 |
| Hypertension | 1,669 (16%) | 337 (12%) | 388 (14%) | 424 (18%) | 520 (21%) | <0.001 |
| Diabetes mellitus | 1,899 (17%) | 153 (5.7%) | 283 (8.8%) | 485 (18%) | 978 (37%) | <0.001 |
| Antihyperlipidemic agents | 55 (0.5%) | 4 (<0.1%) | 16 (0.8%) | 12 (0.6%) | 23 (0.6%) | 0.022 |
| Antidiabetic agents | 193 (1.7%) | 14 (0.4%) | 27 (1.0%) | 48 (1.6%) | 104 (4.1%) | <0.001 |
| Pain | 2,344 (28%) | 535 (24%) | 558 (26%) | 575 (27%) | 676 (33%) | <0.001 |

All values are presented as mean±SD, or counts (weighted, proportion)

PIR: poverty income ratio; HbA1C: hemoglobin type A1C; CVD: cardiovascular disease; CHD: coronary heart disease

**Table S2** Baseline characteristics of participants with chest pain, according to the TyG index

| **Characteristic** | **Overall** | **Q1 (n = 535)** | **Q2 (n = 558)** | **Q3 (n = 575)** | **Q4 (n = 676)** | **P-value** |
| --- | --- | --- | --- | --- | --- | --- |
|  | **(n = 2334)** | **(6.79-8.36)** | **(8.37-8.75)** | **(8.76-9.18)** | **(9.19-13.40)** |  |
| Age, years | 57 (12) | 55 (12) | 57 (12) | 59 (11) | 58 (12) | <0.001 |
| Sex |  |  |  |  |  | <0.001 |
| male | 1,169 (48%) | 228 (39%) | 285 (50%) | 283 (46%) | 373 (56%) |  |
| female | 1,175 (52%) | 307 (61%) | 273 (50%) | 292 (54%) | 303 (44%) |  |
| Race |  |  |  |  |  | <0.001 |
| Mexican American | 330 (4.7%) | 39 (2.6%) | 71 (4.0%) | 87 (5.3%) | 133 (6.6%) |  |
| Other Hispanic | 131 (3.6%) | 19 (2.9%) | 30 (3.0%) | 46 (5.2%) | 36 (3.1%) |  |
| Non-Hispanic White | 1,306 (76%) | 260 (69%) | 316 (78%) | 327 (77%) | 403 (78%) |  |
| Non-Hispanic Black | 460 (11%) | 195 (22%) | 105 (9.4%) | 87 (7.9%) | 73 (6.0%) |  |
| Other race | 117 (5.1%) | 22 (3.2%) | 36 (6.0%) | 28 (4.9%) | 31 (6.2%) |  |
| Education |  |  |  |  |  | <0.001 |
| Under high school | 374 (9.2%) | 64 (7.4%) | 87 (9.0%) | 73 (9.1%) | 150 (11%) |  |
| High school or equivalent | 914 (38%) | 167 (29%) | 213 (36%) | 239 (42%) | 295 (45%) |  |
| Above high school | 1,056 (53%) | 304 (64%) | 258 (55%) | 263 (49%) | 231 (45%) |  |
| Family PIR | 3.00 (1.63) | 3.14 (1.66) | 3.02 (1.59) | 2.97 (1.62) | 2.88 (1.64) | 0.071 |
| BMI, kg/m^2^ | 30 (6) | 27 (6) | 29 (6) | 30 (6) | 32 (6) | <0.001 |
| Smoking status |  |  |  |  |  | 0.015 |
| Current smoker | 541 (24%) | 122 (21%) | 125 (26%) | 127 (24%) | 167 (24%) |  |
| Former smoker | 812 (34%) | 158 (31%) | 174 (30%) | 211 (36%) | 269 (41%) |  |
| Never smoker | 991 (42%) | 255 (48%) | 259 (45%) | 237 (41%) | 240 (35%) |  |
| Total cholesterol, mg/dL | 201 (44) | 187 (40) | 193 (36) | 202 (40) | 218 (51) | <0.001 |
| Triglyceride, mg/dL | 158 (124) | 70 (15) | 106 (16) | 149 (29) | 288 (170) | <0.001 |
| HbA1C, % | 5.77 (1.05) | 5.46 (0.52) | 5.53 (0.51) | 5.71 (0.73) | 6.32 (1.62) | <0.001 |
| Fasting Glucose, mg/dL | 110 (35) | 95 (11) | 102 (14) | 108 (22) | 132 (56) | <0.001 |
| Insulin, uU/mL | 13 (15) | 9 (8) | 11 (8) | 14 (24) | 17 (13) | <0.001 |
| TyG index | 8.85 (0.66) | 8.08 (0.24) | 8.57 (0.11) | 8.95 (0.12) | 9.68 (0.50) | <0.001 |
| CVD | 751 (27%) | 143 (23%) | 167 (24%) | 190 (29%) | 251 (32%) | 0.005 |
| Heart failure | 237 (8.1%) | 39 (6.4%) | 49 (5.8%) | 62 (9.3%) | 87 (10%) | 0.030 |
| CHD | 328 (13%) | 60 (11%) | 70 (10%) | 88 (15%) | 110 (14%) | 0.060 |
| angina | 287 (11%) | 43 (8.4%) | 68 (10%) | 81 (14%) | 95 (13%) | 0.071 |
| Heart attack | 377 (14%) | 71 (11%) | 87 (13%) | 88 (13%) | 131 (17%) | 0.2 |
| Stroke | 197 (6.5%) | 41 (6.4%) | 46 (6.4%) | 50 (6.0%) | 60 (7.1%) | >0.9 |
| Hypertension | 461 (16%) | 97 (13%) | 98 (14%) | 103 (16%) | 163 (20%) | 0.061 |
| Diabetes mellitus | 610 (19%) | 55 (9.1%) | 88 (9.7%) | 144 (19%) | 323 (37%) | <0.001 |
| Antihyperlipidemic agents | 23 (0.7%) | 1 (<0.1%) | 7 (0.8%) | 5 (1.1%) | 10 (0.8%) | 0.2 |
| Antidiabetic agents | 61 (1.7%) | 4 (0.5%) | 5 (0.5%) | 17 (1.3%) | 35 (4.2%) | <0.001 |

All values are presented as mean±SD, or counts (weighted, proportion)

PIR: poverty income ratio; HbA1C: hemoglobin type A1C; CVD: cardiovascular disease; CHD: coronary heart disease

**Table S3** Baseline characteristics of participants without chest pain, according to the TyG index

| **Characteristic** | **Overall** | **Q1 (n= 1549)** | **Q2 (n = 1526)** | **Q3 (n = 1508)** | **Q4 (n = 1408)** | **P-value** |
| --- | --- | --- | --- | --- | --- | --- |
|  | **(n = 5991)** | **(6.79-8.36)** | **(8.37-8.75)** | **(8.76-9.18)** | **(9.19-13.40)** |  |
| Age, years | 57 (12) | 54 (12) | 57 (12) | 58 (12) | 58 (12) | <0.001 |
| Sex |  |  |  |  |  | <0.001 |
| male | 3,001 (48%) | 664 (38%) | 761 (48%) | 774 (50%) | 802 (58%) |  |
| female | 2,990 (52%) | 885 (62%) | 765 (52%) | 734 (50%) | 606 (42%) |  |
| Race |  |  |  |  |  | <0.001 |
| Mexican American | 995 (5.6%) | 161 (3.6%) | 222 (5.2%) | 262 (5.8%) | 350 (8.4%) |  |
| Other Hispanic | 422 (3.3%) | 86 (2.4%) | 97 (2.8%) | 130 (4.7%) | 109 (3.7%) |  |
| Non-Hispanic White | 3,125 (76%) | 804 (76%) | 804 (76%) | 816 (76%) | 701 (77%) |  |
| Non-Hispanic Black | 1,102 (9.3%) | 407 (13%) | 316 (10%) | 210 (7.1%) | 169 (5.3%) |  |
| Other race | 347 (5.5%) | 91 (4.4%) | 87 (5.7%) | 90 (6.2%) | 79 (6.0%) |  |
| Education |  |  |  |  |  | <0.001 |
| Under high school | 876 (6.7%) | 149 (4.3%) | 203 (6.1%) | 242 (7.7%) | 282 (9.6%) |  |
| High school or equivalent | 2,264 (34%) | 541 (29%) | 569 (34%) | 579 (35%) | 575 (41%) |  |
| Above high school | 2,851 (59%) | 859 (67%) | 754 (60%) | 687 (57%) | 551 (49%) |  |
| Family PIR | 3.30 (1.57) | 3.49 (1.54) | 3.36 (1.58) | 3.17 (1.57) | 3.14 (1.58) | <0.001 |
| BMI, kg/m^2^ | 28.8 (6.4) | 26.4 (5.7) | 28.5 (6.6) | 29.7 (6.0) | 31.2 (6.2) | <0.001 |
| Smoking status |  |  |  |  |  | 0.003 |
| Current smoker | 1,054 (18%) | 251 (16%) | 265 (16%) | 263 (19%) | 275 (20%) |  |
| Former smoker | 1,910 (31%) | 449 (29%) | 470 (30%) | 500 (31%) | 491 (34%) |  |
| Never smoker | 3,027 (51%) | 849 (55%) | 791 (54%) | 745 (49%) | 642 (46%) |  |
| Total cholesterol, mg/dL | 205 (41) | 195 (35) | 202 (36) | 209 (42) | 219 (50) | <0.001 |
| Triglyceride, mg/dL | 145 (141) | 68 (15) | 105 (16) | 150 (26) | 288 (248) | <0.001 |
| HbA1C, % | 5.68 (0.87) | 5.39 (0.45) | 5.54 (0.54) | 5.67 (0.65) | 6.22 (1.42) | <0.001 |
| Fasting Glucose, mg/dL | 107 (29) | 95 (11) | 102 (14) | 107 (18) | 129 (50) | <0.001 |
| Insulin, uU/mL | 12 (10) | 8 (6) | 10 (7) | 13 (9) | 18 (15) | <0.001 |
| TyG index | 8.75 (0.63) | 8.05 (0.24) | 8.56 (0.11) | 8.96 (0.12) | 9.64 (0.49) | <0.001 |
| CVD | 588 (7.4%) | 111 (4.6%) | 143 (7.0%) | 161 (9.0%) | 173 (9.9%) | <0.001 |
| Heart failure | 143 (1.8%) | 24 (0.9%) | 34 (1.5%) | 30 (1.6%) | 55 (3.3%) | <0.001 |
| CHD | 201 (2.7%) | 33 (1.5%) | 49 (2.7%) | 57 (3.1%) | 62 (3.7%) | 0.004 |
| angina | 76 (1.1%) | 9 (0.3%) | 21 (1.4%) | 21 (1.0%) | 25 (2.0%) | 0.002 |
| Heart attack | 173 (2.2%) | 29 (1.1%) | 44 (2.6%) | 47 (2.6%) | 53 (3.0%) | 0.003 |
| Stroke | 242 (3.0%) | 57 (2.3%) | 56 (2.6%) | 64 (3.8%) | 65 (3.6%) | 0.059 |
| Hypertension | 1,208 (16%) | 240 (12%) | 290 (15%) | 321 (18%) | 357 (21%) | <0.001 |
| Diabetes mellitus | 1,289 (15%) | 98 (4.6%) | 195 (8.5%) | 341 (17%) | 655 (36%) | <0.001 |
| Antihyperlipidemic agents | 32 (0.4%) | 3 (<0.1%) | 9 (0.8%) | 7 (0.4%) | 13 (0.5%) | 0.11 |
| Antidiabetic agents | 132 (1.7%) | 10 (0.4%) | 22 (1.2%) | 31 (1.7%) | 69 (4.0%) | <0.001 |
| All values are presented as mean±SD, or counts (weighted, proportion)  PIR: poverty income ratio; HbA1C: hemoglobin type A1C; CVD: cardiovascular disease; CHD: coronary heart disease | | | | | | |

**
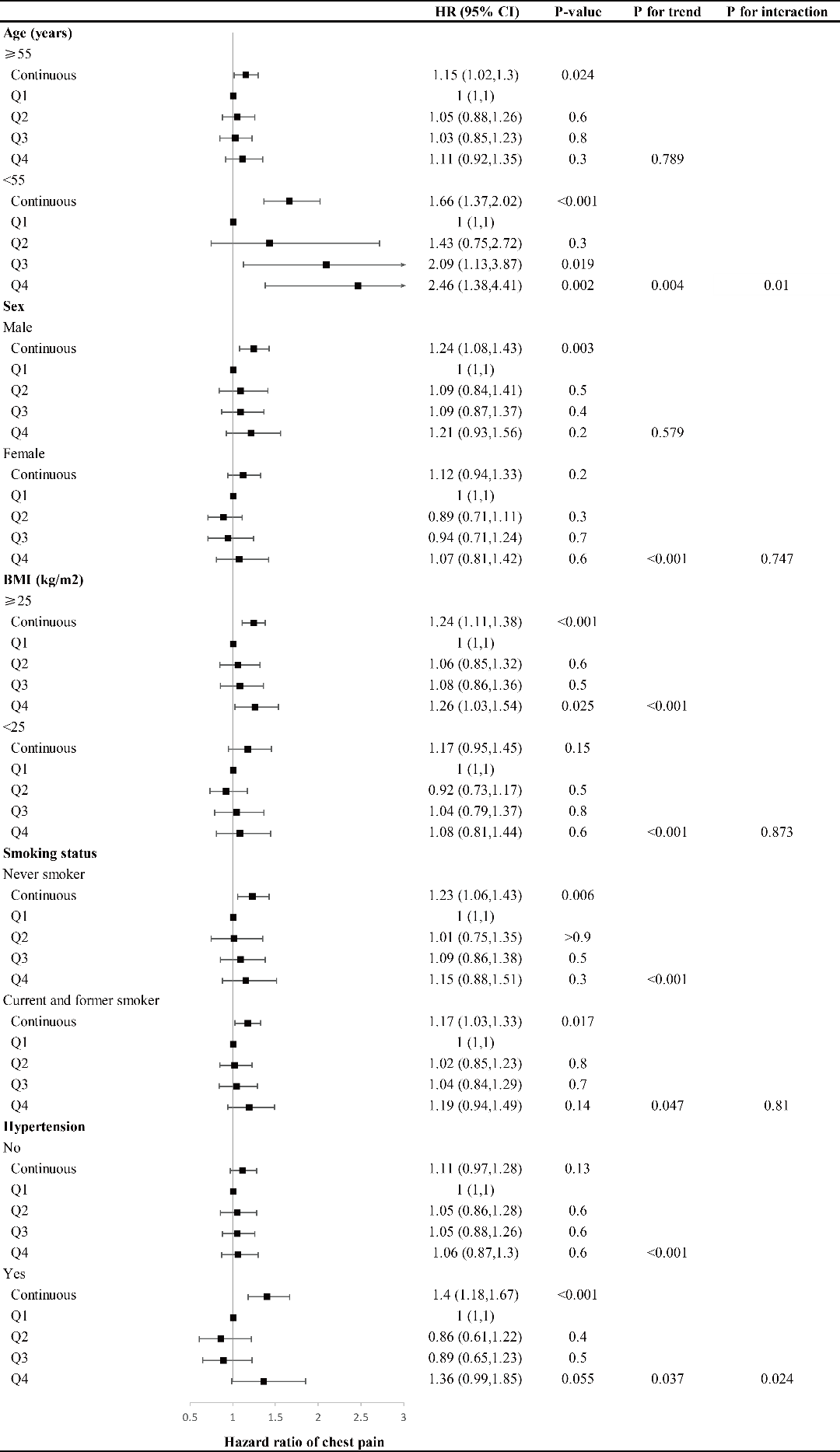
**

**Fig. S1** Subgroup analysis of association between the TyG index and all-cause mortality in all the participants. Adjusted for age, sex, BMI, races, education, smoking status, total cholesterol, and hypertension, heart failure, coronary heart disease, angina, heart attack, stroke, antihyperlipidemic agents, and antidiabetic agents. HR: Hazard ratio, CI: confidence interval.

**
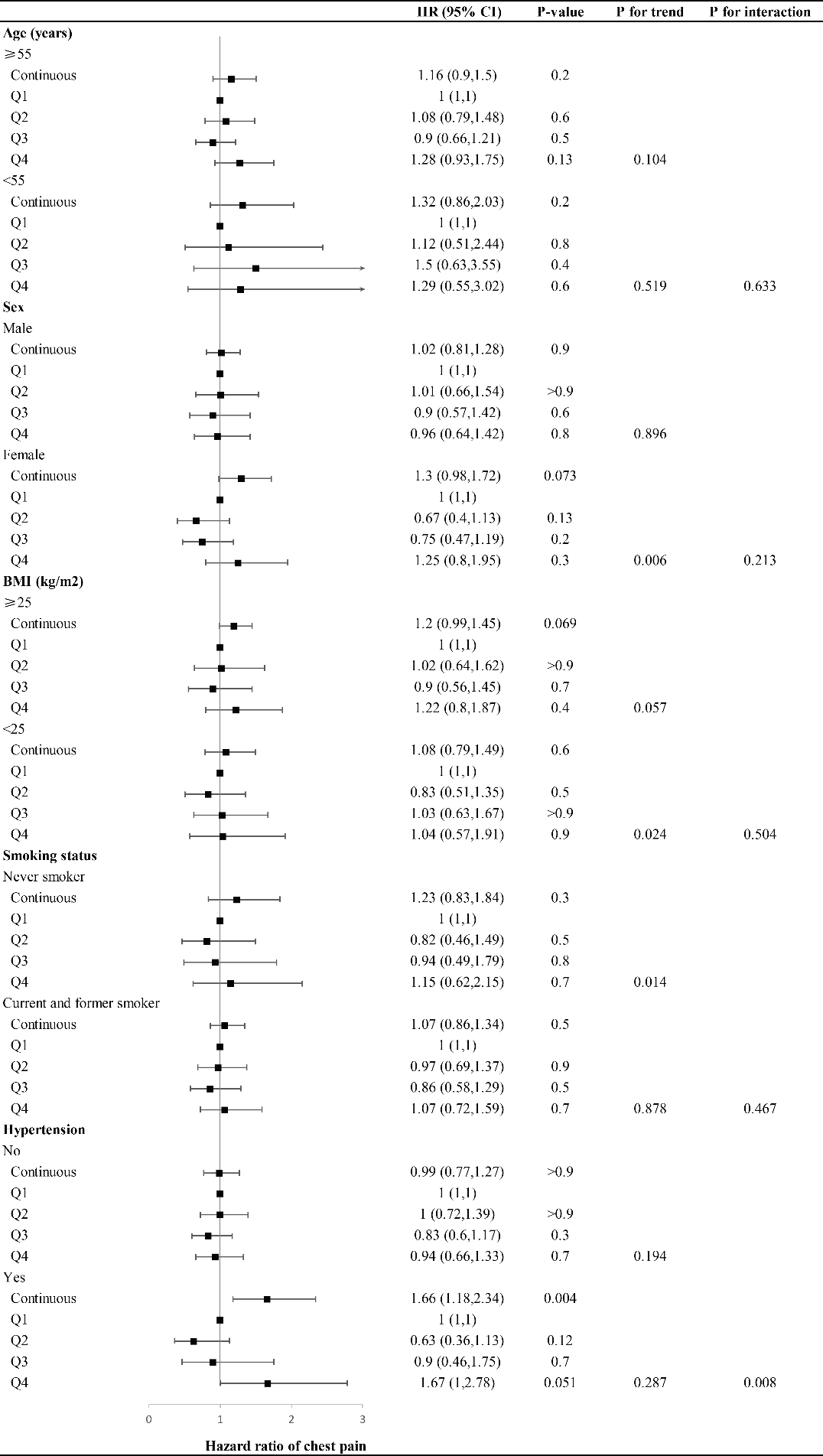
**

**Fig. S2** Subgroup analysis of association between the TyG index and all-cause mortality in participants with chest pain. Adjusted for age, sex, BMI, races, education, smoking status, total cholesterol, and hypertension, heart failure, coronary heart disease, angina, heart attack, stroke, antihyperlipidemic agents, and antidiabetic agents. HR: Hazard ratio, CI: confidence interval.

**
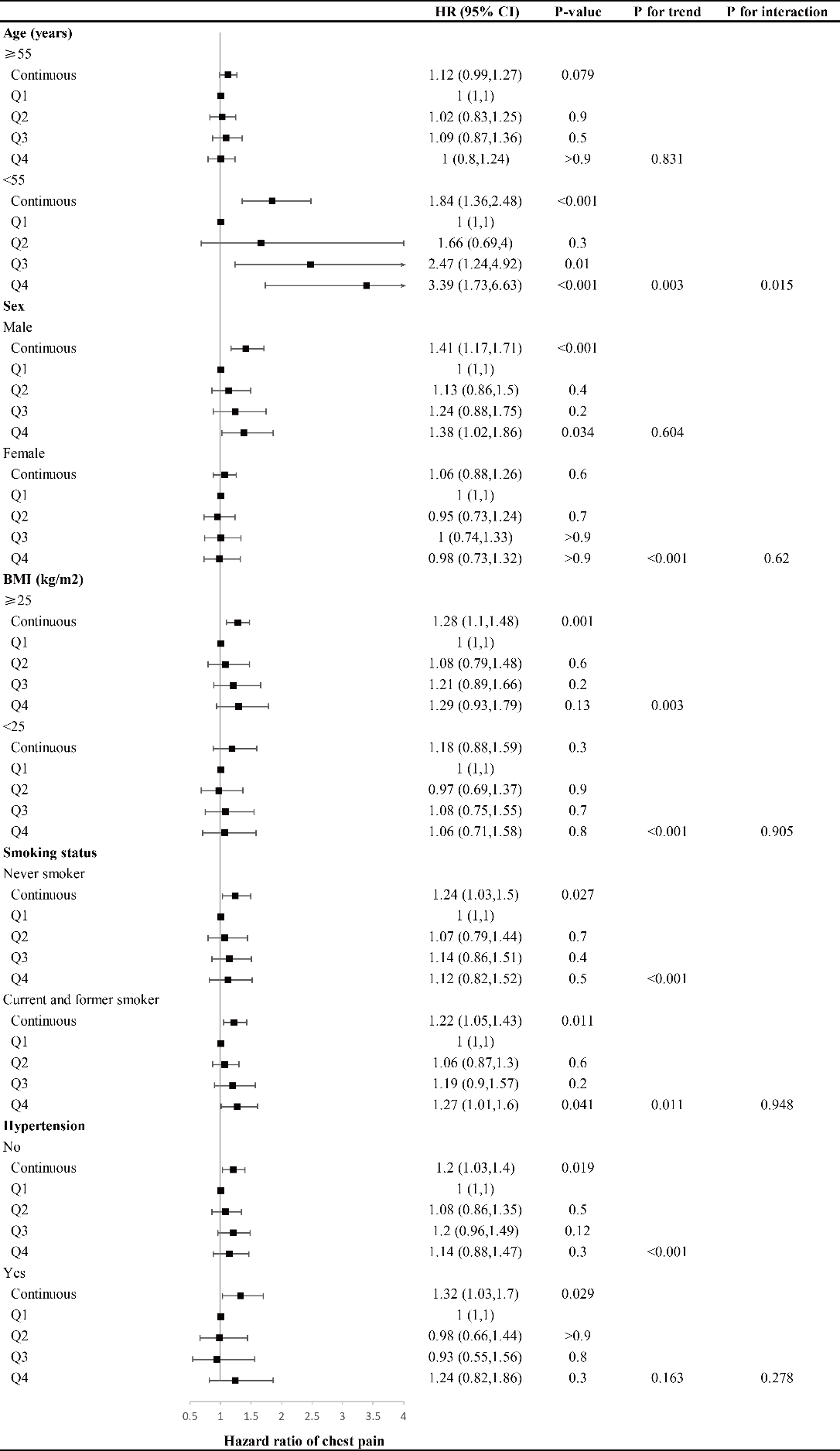
**

**Fig. S3** Subgroup analysis of association between the TyG index and all-cause mortality in participants without chest pain. Adjusted for age, sex, BMI, races, education, smoking status, total cholesterol, and hypertension, heart failure, coronary heart disease, angina, heart attack, stroke, antihyperlipidemic agents, and antidiabetic agents. HR: Hazard ratio, CI: confidence interval.
